# Odor and Constituent Odorants of HDPE–Lignin Blends of Different Lignin Origin

**DOI:** 10.3390/polym14010206

**Published:** 2022-01-05

**Authors:** Bianca Lok, Gunnar Mueller, Johannes Ganster, Jens Erdmann, Andrea Buettner, Philipp Denk

**Affiliations:** 1 Fraunhofer Institute for Process Engineering and Packaging IVV, Giggenhauser Straße 35, 85354 Freising, Germany; bianca.lok@ivv.fraunhofer.de (B.L.); andrea.buettner@ivv.fraunhofer.de (A.B.); 2 Chair of Aroma and Smell Research, Department of Chemistry and Pharmacy, Friedrich-Alexander-Universität Erlangen-Nürnberg, Henkestraße 9, 91054 Erlangen, Germany; 3 Fraunhofer Institute for Applied Polymer Research IAP, Geiselbergstraße 69, 14476 Potsdam, Germany; gunnar.mueller@iap.fraunhofer.de (G.M.); johannes.ganster@iap.fraunhofer.de (J.G.); jens.erdmann@iap.fraunhofer.de (J.E.)

**Keywords:** biomaterials, eco-design, gas chromatography, olfactometry, plastics, polyolefin, smell, sustainability

## Abstract

The still-rising global demand for plastics warrants the substitution of non-renewable mineral oil-based resources with natural products as a decisive step towards sustainability. Lignin is one of the most abundant natural polymers and represents an ideal but hitherto highly underutilized raw material to replace petroleum-based resources. In particular, the use of lignin composites, especially polyolefin–lignin blends, is currently on the rise. In addition to specific mechanical property requirements, a challenge of implementing these alternative polymers is their heavy odor load. This is especially relevant for lignin, which exhibits an intrinsic odor that limits its use as an ingredient in blends intended for high quality applications. The present study addressed this issue by undertaking a systematic evaluation of the odor properties and constituent odorants of commercially available lignins and related high-density polyethylene (HDPE) blends. The potent odors of the investigated samples could be attributed to the presence of 71 individual odorous constituents that originated primarily from the structurally complex lignin. The majority of them was assignable to six main substance classes: carboxylic acids, aldehydes, phenols, furan compounds, alkylated 2-cyclopenten-1-ones, and sulfur compounds. The odors were strongly related to both the lignin raw materials and the different processes of their extraction, while the production of the blends had a lower but also significant influence. Especially the investigated soda lignin with *hay*- and *honey*-like odors was highly different in its odorant composition compared to lignins resulting from the sulfurous kraft process predominantly characterized by *smoky* and *burnt* odors. These observations highlight the importance of sufficient purification of the lignin raw material and the need for odor abatement procedures during the compounding process. The molecular elucidation of the odorants causing the strong odor represents an important procedure to develop odor reduction strategies.

## 1. Introduction

The widespread global use of non-renewable, petroleum-based commodities could be abated, at least in part, by their replacement with materials derived from natural resources. As the most abundant bio-based polymer in nature [1], lignocellulose materials represent a viable and sustainable alternative with the benefit of being carbon-neutral and commercially available at low cost [2,3]. Lignin represents one of the three main components of lignocellulose mainly sourced from softwood, hardwood, or grass, but has hitherto been widely considered as an inferior by-product or waste material of pulping [4,5]. Lignin is a hetero-aromatic polymer that exhibits heavily diverse chemical cross-linkage, thereby ensuring structural integrity in plants [6]. Due to its complex composition comprising diverse monomer structures and modes of conjugation, the determination of the exact structure is challenging. Generally, lignin primarily consists of three different cinnamyl alcohol-derivative monomers forming various phenylpropanoid structures, depending on the plant species, and forms characteristic species-specific patterns [7,8].

The industrial isolation of technical lignin is currently undertaken mainly using either the kraft or the sulfite process, both of which utilize sulfurous reactants. While the latter relies on the reaction between lignin and free sulfurous acid, the kraft process uses sodium hydroxide and sodium sulfide for the progressive breakdown to lower molecular weight fragments under strong alkaline conditions [8,9]. A third method often applied for non-wood-based biomaterials is the sulfur-free soda process that utilizes sodium hydroxide. This process has been reported to yield technical lignin of lower molecular weight and higher purity [7,8]. The physicochemical properties of technical lignin strongly depend on the extraction process and the organic source of the lignin, but in general they hold great potential [10]. Although a large proportion is nowadays discarded, mainly combusted, lignin represents the only sustainable large-volume resource to provide aromatic monomers [11]. Further, beyond its current use in resins, adhesives and foams, the potential fields of application of technical lignin are manifold, including its utilization in polymer blends, especially polyolefin blends [4,12].

Used as either a low-cost filling material or formulation component, lignin can be exploited as part of polyolefin composite materials in the production of molding parts for various purposes [10,13,14,15,16,17]. To meet industrial needs, however, a critical and problematic parameter is most often the insufficient compatibility of lignin with the respective polymer [12,18,19]. Poor interaction between the polar lignin and hydrophobic polymers can result in diminished mechanical properties, such as material embrittlement and reduced tensile strength and elongation [18,20]. With the goal of enhancing its compatibility, current strategies comprise the addition of coupling agents or the chemical modification of lignin itself [12,21]. In this regard, the combination of thermoplastics with special low-molecular fractions of lignin is one of the most progressive methods so far [17]. Furthermore, by improving the homogeneity of the polyolefin–lignin composite structure, i.e., by dispersing the lignin particles below a certain threshold, the embrittlement effect by lignin itself can be overcome. This patented technology has led to the implementation of composites with mechanical properties equal or even higher than the pure polyolefins [22].

Beyond the mechanical properties, the odor load of lignin is a critical factor affecting the quality characteristics of the ensuing materials, and consequently the market and consumer acceptance of such composites [23]. While currently being a decisive parameter with the potential of limiting broader applications, unwanted or even offensive odors have rarely been thoroughly evaluated in composite materials but have repeatedly been reported during the production process of lignin—either as malodors from the raw kraft liquor or as disturbing emissions from kraft mills [24,25,26]. Volatile sulfur compounds, foremost methanethiol, hydrogen sulfide, dimethyl sulfide and dimethyl disulfide, along with other phenolic volatile organic compounds (VOCs) have been associated with the perceived odors, in particular the quantitatively dominating compounds guaiacol and vanillin. Yet, intense contributors to odor nuisances are often found as trace compounds, whereby their impact is driven by their extremely low odor thresholds [27]. Accordingly, a combinatory approach of advanced analytics and human sensory evaluation is commonly required for the targeted elucidation of such potent smell substances. Referring to technical lignins, several approaches have targeted a deodorization by reducing low-molecular, phenolic VOCs and sulfur compounds through enzymatic treatment followed by subsequent purification techniques, for example, extraction with supercritical CO_2_ [23,28].

In terms of lignin blends, however, there is still a knowledge gap on the substances causing off-odor, which we aim to close in the present study by a comprehensive elucidation of odor-active compounds in lignin composites. To this aim, human-sensory evaluation was combined with analytical structure elucidation comprising two-dimensional gas chromatography-mass spectrometry/olfactometry (2D-GC-MS/O) to assign specific odor characteristics with their causative molecules. The scope of the study reaches from the lignin raw material itself to corresponding HDPE-lignin blends, whereas the associated odors were investigated with respect to the applied processing parameters. Diverse lignins of different origin and quality were examined in relation to their influence on the odor. Using this systematic approach, we aim to provide new insights into the formation pathways of odor that will allow for the development of odor reduction strategies.

## 2. Materials and Methods

### 2.1. Sample Material

#### 2.1.1. Origin and Characteristics of Raw Materials

For the production of the HDPE–lignin blends, three commercially available lignins resulting from different raw materials and processing methods were used (see Table 1). The lignins were obtained in 25 kg bags and dried for 24 h at 60 °C in vacuum over silica gel to reduce the water content to <1.5 wt%. Two of the investigated lignins resulted from kraft processed softwood, whereas one of these lignins originated from pinewood only and was specified as highly purified. The third lignin was made out of wheat straw and grass and resulted from the sulfur-free soda process.

Besides, customary HDPE intended for use in injection molding applications with a narrow molecular weight distribution was selected. HDPE was chosen with regard to the production of highly functional polymers as especially HDPE–lignin composites feature valuable mechanical properties. Composites made with other (bio-based) polymers do not (yet) comply with requirements regarding material properties and price. The focus was therefore on finding short-term realizable solutions for the extensive material use and application of lignin. The virgin HDPE as well as the HDPE-based compatibilizer Fusabond E-MB 100D (DuPont) were used without further pre-treatment.

#### 2.1.2. Production of HDPE-Lignin Blends

The HDPE–lignin blends were prepared by compounding HDPE M80064, Fusabond E-MB 100D, and the respective lignin (a ratio of 63:7:30 by weight) in a kneader (W 350, Brabender) by preheating the kneading chamber up to 210 °C and then charging the respective components consecutively starting with HDPE M80064, afterwards the compatibilizer. The respective lignin was added in the final step upon complete melting of the HDPE components. The resulting mixture was then kneaded for three more minutes under an atmosphere of nitrogen. The melt temperature was 195 °C. Upon cooling the blend to room temperature, the material was further cooled by liquid nitrogen and then milled in a cutting mill (Alpine Rotoplex 10/6).

### 2.2. Sensory Evaluation

Sensory evaluations were performed by five trained panelists (3 female, 2 male) from Fraunhofer IVV (Freising, Germany). Weekly training of the assessors was based on the recognition and description of approximately 150 different odorants based on an in-house odor language, which were presented in the form of odorant pens. Panelists did not exhibit any known illness or olfactory dysfunction at the time of examination.

Following the DIN 10967-2 (investigation of profiles—consensus profile and free choice profiling), the samples were presented in sealable, odorless plastic cups, randomized and encoded with three-digit numbers. In a first session, all HDPE–lignin blends were presented to the panel simultaneously, followed by a second session comprising all pure lignins. In an initial group discussion, the sensory attributes were collected, discussed and defined, which allowed a maximum differentiation between the different sample materials. The following attributes were chosen by the panel: *sulfuric*, *smoked ham-like/clove-like*, *burnt/charcoal-like*, *butter-like*, *vanilla-like*, *hay-like*, and *honey-like*. Immediately after the group discussion, assessors rated the intensity of each attribute in the samples individually, using a scale from 0 (no perception) to 10 (strong perception). Statistical interpretation of the obtained results was performed by a Generalized Procrustes Analysis (GPA) using the software XLSTAT.

### 2.3. Instrumental–Olfactometric Analyses

#### 2.3.1. Solvent Extraction and Isolation of Volatiles

Volatiles were extracted from 2 g (±0.1 g) of each sample material (pure lignin powders and HDPE–lignin blends, respectively) using 50 mL of dichloromethane (DCM) by stirring for 30 min at room temperature. After filtration through filter paper, the volatile fraction, including the odor-active compounds, was carefully isolated from the extract by performing solvent-assisted flavor evaporation (SAFE) under high vacuum while the apparatus temperature was kept at 55 °C [29]. Subsequently, the obtained distillates were dried over anhydrous sodium sulfate, again filtered and concentrated at 50 °C to a total volume of ~100 μL by Vigreux distillation (50 cm × 1 cm i.d.) and micro distillation [30]. Applying the identical work-up, blank samples were prepared with 50 mL DCM.

Details of chemicals, solvents and especially of reference compounds used for the identification of detected odorants are provided in Appendix A.

#### 2.3.2. GC-O and Odor Extract Dilution Analysis (OEDA)

For the assignment of relative odor intensities to single odor-active regions perceivable during gas chromatographic analyses, the original distillates (Section 2.3.1), representing odor dilution (OD)-factor 1, were diluted stepwise (1:3; *v*/*v*) with DCM resulting in solutions corresponding to OD-factors in a 3^n^ series up to 2187. Performing GC-O analyses of all obtained dilutions enabled the assignment and comparison of OD-factors of single odor-active regions. Accordingly, higher OD-factors imply a potentially higher relevance for the overall odor of a sample (odor extract dilution analysis [OEDA], [31,32]).

Performed in triplicate by three different assessors, the original distillates were analyzed by GC-O using a Trace GC Ultra (Thermo Fisher Scientific GmbH, Dreieich, Germany) using the capillary column DB-FFAP (30 m × 0.32 mm, 0.25 μm film thickness; J&W Scientific, Agilent Technologies, Waldbronn, Germany). Further, the system included a pre-column (uncoated, deactivated fused silica capillary, 5 m × 0.32 mm), as well as a flame ionization detector (FID, 250 °C) and an odor detection port (ODP, 250 °C), which were both connected to the end of the main column via Y-type glass splitter and two uncoated, deactivated fused silica capillaries (0.5 m × 0.2 mm). This setting ensured a split in a 1:1 volume ratio and simultaneous detection at both detectors. Injection of 2 μL aliquots was performed manually at 40 °C (cold on-column technique; 40 °C held for 2 min). The oven temperature was then raised at 8 °C/min to 230 °C, holding the final temperature for further 5 min. With a flow rate of 2.2 mL/min, helium was used as carrier gas. Analysis of each dilution up to OD-factor 2187 was performed equally. The original distillates were further analyzed with the identical set-up using a capillary column of different polarity (DB-5, 30 m × 0.32 mm, 0.25 μm film thickness; J&W Scientific, Agilent Technologies, Waldbronn, Germany) with a final oven temperature of 250 °C.

Additionally, a homologous series of *n*-alkanes was analyzed using the exact same parameters as described above, to allow the calculation of linear retention indices (RIs) on both capillary columns of different polarity [33]. Along with the perceived odor quality at the ODP, the determined RIs allowed the initial identification of odorants. This tentative identification was then further substantiated by the following analytical steps: record of the respective mass spectrometric data and comparison of all identification parameters to those of reference compounds by performing GC-MS/O and 2D-GC-MS/O for each substance individually (Section 2.3.3). The compiled data then formed the basis for the final unequivocal identification of the detected odorants.

#### 2.3.3. GC-MS/O and Two-Dimensional GC-MS/O

GC-MS/O analyses were performed comparable to GC-O with the following differences only: instead of an FID, a DSQ II MS (Thermo Fisher Scientific GmbH, Dreieich, Germany) was used as a detector and sample aliquots were injected by a multipurpose autosampler (MPS 2, Gerstel GmbH & Co. KG, Mülheim an der Ruhr, Germany; see [34] for a detailed list of instrument parameters). Mass spectra (if obtained) were generated in electron ionization (EI) *full scan* mode (70 eV) over an *m/z* range of 35–399).

For ultra-trace compounds or odorants that co-eluted heavily with other (odorless) volatiles, additional 2D-GC-MS/O analyses were required to obtain the required mass spectrometric data. The heart-cut system comprised two Varian CP-3800 GCs (Agilent Technologies, Waldbronn, Germany) connected via a cryotrap system (CTS 1, Gerstel GmbH & Co. KG, Mülheim an der Ruhr, Germany) cooled to −100 °C with liquid nitrogen. Sample aliquots of 4 μL of the original distillates (OD-factor 1) were automatically injected (MPS 2XL multipurpose sampler; Gerstel) to a DB-FFAP capillary column in the first oven starting at 40 °C for 2 min. The temperature was then raised at 8 °C/min to 230 °C and held for 5 min, while the helium carrier gas was kept at 9.0 mL/min. Similar to the GC-O system, the effluent of the first dimension was split via a Y-type glass splitter to two detectors (FID and ODP, at 250 °C and 270 °C, respectively). For the isolation and the transfer of the analytes of interest into the second dimension by means of a multi-column switching system (MCS2, also Gerstel), cryotrapping (−100 °C) was followed by thermodesorption (250 °C). Equipped with a capillary column of different polarity (DB-5), the initial temperature of the second GC oven (40 °C) was raised (8 °C/min) to 250 °C and held for 1 min. Again, splitting the final effluent via a Y-type glass splitter allowed the simultaneous record of the respective odor quality (ODP; 270 °C) and the corresponding mass spectra (EI, 70 eV, *m/z* 35–399; Saturn 2200 ion trap MS, Agilent Technologies, Waldbronn, Germany).

## 3. Results

### 3.1. Sensory Evaluation

**Figure 1:**
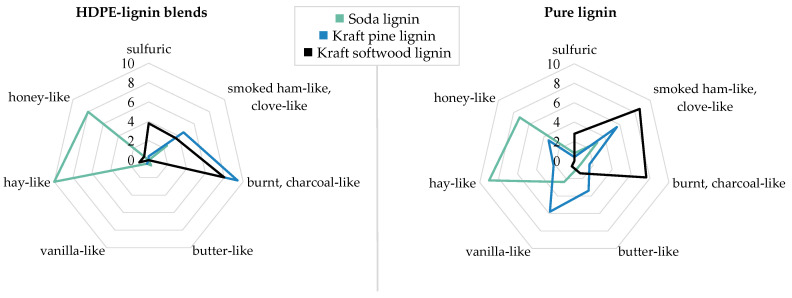
Descriptive odor profiles of (**right**) the pure lignins and (**left**) corresponding HDPE blends (mean ratings obtained from the sensory evaluation, *n* = 5, scale from no perception (0) to strong perception (10)).

Sensory evaluation initially revealed a high odor load of both the HDPE-lignin blends as well as the corresponding lignins that were used as raw material. Seven odor attributes, as chosen by the trained sensory panel, served for the description of the smell character, and allowed a clear sensory distinction of the investigated sample materials. In addition to the odor impressions *sulfuric*, *smoked ham-like/clove-like,* and *burnt/charcoal-like*, the panel agreed on the attributes *butter-like*, *vanilla-like*, *hay-like,* and *honey-like* (Figure 1).

Prominent odor impressions for the soda lignin and the associated HDPE-lignin blend were *hay-like* and *honey-like,* which revealed high mean ratings of 9.0/10.0 (*hay-like*) and 7.2/8.0 (*honey-like*). Apart from *smoked ham-like/clove-like* (3.0/2.4 for the pure lignin and the blend, respectively) and *vanilla-like* (2.4 in the pure lignin), all other attributes were negligible since they were rated with mean ratings below 1.0, thus being not regarded as sufficiently perceivable.

On the contrary, the odor profiles of both kraft HDPE–lignin blends were predominantly perceived as being *burnt/charcoal-like* (mean ratings of 9.4/8.0 for the pine HDPE–lignin blend and the softwood HDPE–lignin blend), followed by a *smoked ham-like/clove-like* odor impression with mean ratings of 4.6/3.6. Except of a *sulfuric* note only perceived in the softwood HDPE–lignin blend (3.8), the odor profiles of both kraft HDPE–lignin blends were very similar but clearly distinguishable from the soda lignin and the corresponding HDPE blend.

The *burnt/charcoal-like* odor was also strongly perceived in the pure kraft softwood lignin (7.6). However, this lignin was additionally characterized by the *smoked ham-like/clove-like* odor, achieving the highest mean rating of 8.6. The intense *smoked ham-like/clove-like* smell character was the main difference in the odor profiles of the pure kraft softwood lignin and the corresponding blend.

In contrast to the kraft softwood lignin, the pure kraft pine lignin was less similar to its corresponding blend. Here, none of the attributes reached ratings in the upper scale range as in case of the other sample materials. In particular, the pure kraft pine lignin was described heterogeneously with ratings for *vanilla-like* (5.8) and *smoked ham-like/clove-like* (5.6), followed by *butter-like* (3.4) and *honey-like* (3.4), as well as *hay-like* (2.2) and *burnt/charcoal-like* (1.6). Interestingly, the *burnt/charcoal-like* odor impression that had been most intensely perceived in the corresponding blend (9.4) was only rated with 1.6 in the pure lignin.

**Figure 2:**
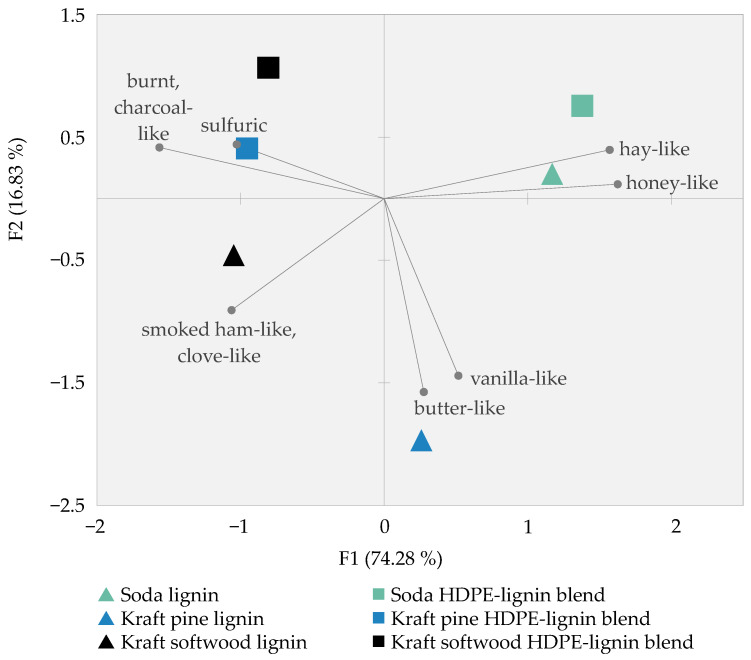
Results of the GPA for the first and second dimension including the odor attributes determined by the panel during sensory evaluation.

Statistical analysis of the sensory results illustrated the previous findings based on the mean ratings for the single odor attributes. Performance of a Generalized Procrustes Analysis (GPA) revealed that 74.3% of the variability of the samples based on the perceived odor was concentrated on the first axis and 16.8% on the second axis (Figure 2). In detail, both the pure soda lignin as well as its corresponding HDPE blend clearly separated on the map and formed one cluster, emphasizing their similarity in odor mainly described as *hay-like* and *honey-like* as seen by the close proximity to those two attributes. Further, the panel differentiated well this soda lignin cluster from all other sample materials based on kraft lignin. In case of the latter, the similarity in odor of the kraft lignin HDPE blends was illustrated by the proximity on the map closely located around the attributes *burnt/charcoal-like* and *sulfuric*. Although closely located to both kraft lignin HDPE blends, the pure kraft softwood lignin differed from this cluster due to its strong *smoked ham-like/clove-like* smell, which also explained the location of this sample in the third quadrant. Besides, as visualized by the isolated location to the odor characteristics *butter-like* and *vanilla-like*, the panel differentiated well the pure kraft pine lignin. This emphasized not only the clear distinguishability of the pure kraft pine lignin from all other sample materials but also from its corresponding blend.

### 3.2. Identification of Odor-Active Compounds via Instrumental Analyses

Subsequent to the sensory evaluation, analyses of the solvent distillates of the pure lignins as well as the HDPE blends enabled the elucidation of single odorous substances responsible for the perceived smells. Speaking of all sample materials as a whole, an initial olfactometric screening performed by GC-O led to the detection of a total of 71 odor-active regions (see Table 2). Thereof, 90% were identified on a molecular level by means of GC-MS/O and 2D-GC-MS/O. Assignment of OD-factors by performing OEDA (Section 2.3.2) gave additional information on the relative odor potency of single odorants in the GC-O analyses.

With regard to the pure lignins, the soda lignin represented the sample where the total number of odor-active compounds was the highest. In particular, 49 odorants were detectable in the original distillate, whereas in both kraft lignins, 32 and 48 odorants (pine lignin/softwood lignin) were detected. In the case of the HDPE–lignin blends, 50, 41 and 42 odorants, respectively, were detected in the soda HDPE–lignin blend, in the kraft pine, and the softwood HDPE–lignin blend. All three samples showed comparable numbers of odorants perceived in the highest dilutions (4–5 odorants with OD-factors ≥ 243 per sample). In contrast, in the analyzed sample aliquot of the virgin HDPE pellets, none of the reported odorous substances was detectable.

Due to the number and structural diversity of the detected odorants, individual substance groups will be discussed section-wise, concentrating on six different chemical classes as described in the following. With 54 of the 71 detected odorants assignable to these groups, the specified substance classes thus cover the majority of detected odor-active compounds.

#### 3.2.1. Carboxylic Acids

Comprising a total number of eight compounds, the carboxylic acids represent one important odorant group in this study. In addition to common unbranched short-chain carboxylic acids such as acetic acid (no. 20, Table 2), butanoic acid (no. 29), and pentanoic acid (no. 37), we detected further three methylated carboxylic acids, namely 2-/3-methylbutanoic acid, 3-methylpentanoic acid, and 5-methylhexanoic acid (no. 32, 41, 51). With regard to the pure lignins, the carboxylic acids were perceived with the highest OD-factors in the soda lignin, whereas the level in both kraft lignins was lower yet comparable in intensity. For example, 2-/3-methylbutanoic acid (no. 32) was detected in the soda lignin up to a dilution step that corresponds to OD-factor 81, but only with OD-factors 3 and 9 in the kraft pine and softwood lignin, respectively. In the corresponding blends, only few carboxylic acids were perceived. In particular, none of the carboxylic acids was detected in case of the softwood HDPE–lignin blend.

In contrast to these primarily *cheesy/sweaty* smelling short-chain carboxylic acids, phenylacetic acid (no. 68) and 3-phenylpropanoic acid (no. 70), both characterized by the functional phenyl group and a *honey-like* smell, were perceived the strongest in the pure soda lignin with OD-factors of 243 and 9, respectively. In the kraft lignins, both were detected with 2 to 4 OD-factor steps lower. Comparably, both substances were detected in the soda HDPE–lignin blend but not in both kraft lignin blends.

#### 3.2.2. Saturated and Unsaturated Aldehydes

In total, ten unsaturated and a further three saturated aldehydes of various chain lengths (C_6_–C_11_) could be detected in the samples (no. 4, 8, 10, 16, 18, 22–24, 26, 31, 36, 40, and 43). All of them were perceived in the distillate of the pure soda lignin, some of them up to high OD-factors, such as (*E*)-2-nonenal (no. 24) with the second highest OD-factor of 729, followed by (*E,Z*)-2,6-nonadienal (no. 26) and (*E,E*)-2,4-nonadienal (no. 36) with OD-factors of 243 and 81, respectively. Another five saturated and unsaturated aldehydes, namely hexanal (no. 4), (*Z*)-4-heptenal (no. 8), (*E*)-2-octenal (no. 18), (*Z*)-2-nonenal (no. 23) and (*E*)-2-undecenal, yielded OD-factor 27 in the pure soda lignin. In contrast to the high level of these mainly *fatty* smelling compounds in the pure soda lignin, they were less pronounced in the distillates of both kraft lignins. In particular, none of the saturated and only four and five unsaturated aldehydes were perceived in the pine and the kraft softwood lignin, respectively, with OD-factors no higher than 9.

Comparable to the pure lignins, the soda lignin HDPE blend showed the greatest level of aldehydes, with the highest OD-factor of 81 for (*E*)-2-nonenal, that was similarly detected with OD-factor 81 in the kraft softwood lignin. Other than this, only six unsaturated aldehydes were perceived in both kraft lignin HDPE blends, and these did not exceed OD-factor 9. Accordingly, both kraft lignin HDPE blends were quite divergent from the soda lignin HDPE blend in this respect.

#### 3.2.3. Phenolic Compounds

Characteristic for all samples was the detection of a series of phenolic compounds containing different functional groups such as alkyl, methoxy, vinyl, propenyl, and acetyl moieties (no. 47, 48, 50, 55–60, 62, 64, 69, 71). The majority of these substances were still perceived in the highest dilutions, above all the *smoky, smoked ham-like* smelling guaiacol (no. 47) which was detected in almost all samples with OD-factor 2187. Additionally, the *vanilla-like* smelling vanillin (no. 69) was detected in all distillates with OD-factors equal or higher than 243. Structurally related phenolic compounds with additional functional groups were 2-methoxy-5-methylphenol (no. 50), 2-methoxy-4-vinylphenol (no. 60), 2,6-dimethoxyphenol (no. 62), isoeugenol (no. 64), and 4-acetyl-2-methoxyphenol (no. 71).

In contrast to these phenols containing methoxy groups, the alkylated phenolic compounds showed considerable lower OD-factors and were detected with OD-factors up to 27 only in a few cases, such as 2,6-dimethylphenol (no. 48) in the pure kraft softwood lignin or *p*-cresol (no. 55) in the pure soda lignin. Only the *leather-like* smelling 3-ethylphenol (no. 58) was detected with the second highest OD-factor of 729 in the pure soda lignin, and with OD-factors of 27 and 9 in the kraft softwood and pine lignin, respectively. In the corresponding HDPE-lignin blends, 3-ethylphenol was detected at levels that were 1 to 3 OD-factor steps lower.

#### 3.2.4. Compounds with a Furan-Derived Core Structure

Another important substance class of odorants detected in the sample distillates were compounds with a furan-derived structure. As one characteristic odorous representative, the *caramel-like* smelling furaneol (no. 53) was perceived with high OD-factors of 27 up to 243 in the HDPE–lignin blends. Besides, further three furan-related and sulfur-containing compounds were perceivable in all blends (no. 19, 33, 34). Especially the *roasted coffee bean-like* smelling 2-furfurylthiol (no. 19) and the *broth-like, meat-like* smelling 2-methyl-3-(methyldithio)furan (no. 34) were predominantly detected with OD-factor 81 in all HDPE–lignin blends. Another odor-active region characterized by the same *broth-like, meat-like* smell was associated with the highly odor-potent compound no. 52 that was also detected with OD-factor 81 in the soda HDPE–lignin blend, and with OD-factor 729 in both kraft HDPE–lignin blends. However, the exact molecular structure could not be assigned to a corresponding reference compound as this compound was found to be present at traces only so that no mass spectrum could be recorded for unequivocal identification of the substance. Whereas this unknown odorant together with almost all other furan-related compounds revealed such high OD-factors up to 729 in the blends, substantially lower OD-factors were detected in the pure lignins. Except of the detection of 2-furfurylthiol with OD-factor 27 in the kraft softwood lignin, furan-related compounds did not exceed OD-factor 3 or were not detected at all in the pure lignins.

#### 3.2.5. Alkylated 2-Cyclopenten-1-Ones

Apart from the furan derivatives, three odorous compounds showed a basic structure of a 2-cyclopenten-1-one comprising different alkyl groups. The *caramel-like* smelling 3-ethyl-2-hydroxy-2-cyclopenten-1-one (no. 42) was detected with the highest OD-factor 2187 in the soda and the kraft softwood lignin, and in the kraft pine lignin with OD-factor 243. Cycloten (no. 45) was detected in all pure lignins with comparably high OD-factors. This compound is characterized by its typical *lovage-like* smell and its very similar structure with the sole difference that a methyl group is attached to C3 instead of an ethyl group. A distinctive *caramel-like* smell, comparable to that of 3-ethyl-2-hydroxy-2-cyclopenten-1-one, was perceived for compound no. 46, closely eluting after cycloten on the capillary column DB-FFAP. Based on the recorded mass spectrum showing the typical base fragmentation pattern of both alkylated 2-cyclopenten-1-ones, it can be assumed that compound no. 46 features the same basic molecular structure but contains a different alkyl substituent. Apart from the detection of all three compounds with high OD-factors in the pure lignins, they were also perceived in the distillates of the corresponding HDPE–lignin blends, albeit with one to four OD-steps lower.

#### 3.2.6. Sulfur Compounds

Sulfur compounds made up another representative substance class with a total of 12 constituents detected in the entirety of the sample materials, thereby not including the three sulfurous furan compounds previously discussed (Section 3.2.4). On one hand, linear sulfur containing alkanes were perceivable with a typical *garlic-like, cabbage-like* smell such as dimethyl di-/tri- and tetrasulfide (no. 3, 14, 39), as well as bis(methylthio)methane (no. 9) and 1,1-bis(ethylthio)ethane (no. 17). In fact, dimethyl trisulfide (no. 14) was the most important sulfur compound with OD-factors 27–243 in the HDPE blends, closely followed by dimethyl tetrasulfide (no. 39), with OD-factor 27 in both kraft blends but OD-factor 1 only in the soda HDPE-lignin blend.

On the other hand, structurally diverse sulfur and oxygen containing hydrocarbons were detected (no. 6, 12, 15, 30, 38), along with two unknown but presumably sulfur containing odorants as assumed by their typical *sulfurous* smell perceived by the panelists (no. 5 and 7). Most of them were only detectable with notable OD-factors in the kraft softwood lignin, such as in the case of 4-mercapto-4-methyl-2-pentanone (no. 15) and 2-acetyl-2-thiazoline (no. 38). On the contrary, 4-methoxy-2-methyl-2-butanethiol (no. 6), 1-methoxy-3-methyl-3-pentanethiol (no. 12) and 4-mercapto-2-butanol (no. 30) showed higher OD-factors in the kraft softwood HDPE blend, however, not exceeding OD-factor 9.

The kraft softwood lignin and its corresponding HDPE blend were mostly associated with the detected sulfur compounds, both with regard to number of compounds as well as their related OD-factors. In total, 10 and 8 sulfur compounds, respectively, were perceived in the pure kraft softwood lignin and its blend with OD-factors up to 81. In contrast to that, only two sulfur compounds with low OD-factors only were detected in the distillate of the kraft pine lignin, whereas another four sulfur compounds were perceived in its corresponding blend. In case of the soda HDPE–lignin blend, only dimethyl trisulfide was detected with OD-factor 27. In the corresponding pure soda lignin, no other sulfur compound was detectable.

## 4. Discussion

### 4.1. Correlation of Sensory Results with Analytically Identified Odorants

**Figure 3:**
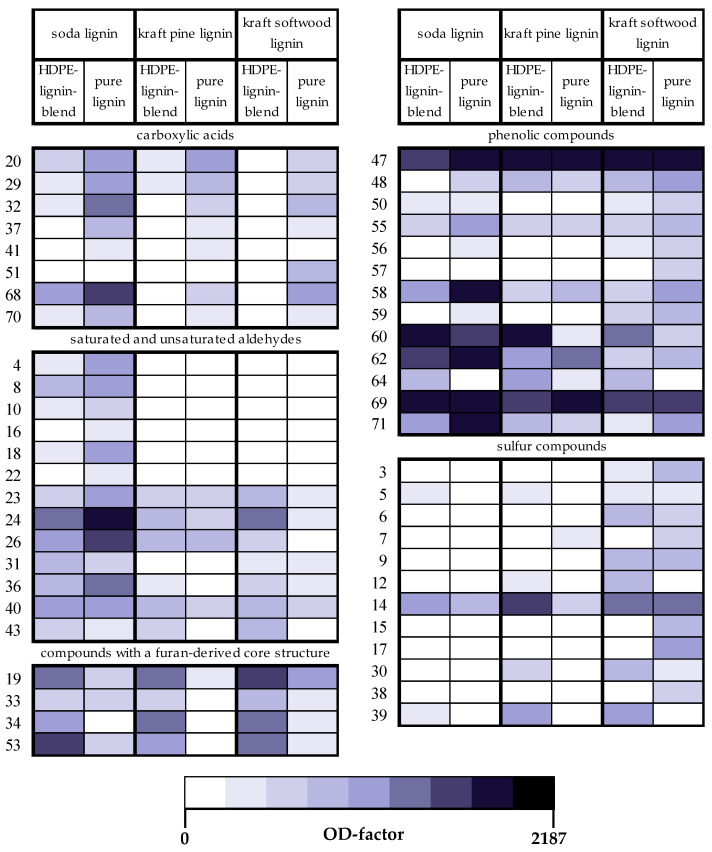
Color-coded illustration of differences in OD-factors of the main chemical classes of odorants (carboxylic acids, aldehydes, compounds with a furan-derived core structure, phenolic and sulfur compounds) detected in distillates of the pure lignins and corresponding HDPE blends. More intense colors represent higher OD-factors and thus possible greater impact on the overall odor.

The odor profiles of the soda lignin and its corresponding HDPE blend were highly comparable, both characterized by the *hay-like* and *honey-like* smell as perceived by the panel. Forming one cluster in the GPA (see Figure 2), both samples were clearly distinguishable from all other samples. Subsequent analytical characterization allowed the elucidation of trends in single odorant groups potentially causing the differences in odor. Together with the carboxylic acids, the large group of aldehydes showed substantially higher OD-factors in the soda lignin than in both kraft lignins. Likewise, much higher values especially of the aldehydes were observed for the soda HDPE–lignin blend compared to both kraft HDPE–lignin blends (see Figure 3). Besides, the detection of considerably fewer sulfur compounds in the soda lignin and its corresponding blend compared to the kraft softwood lignin and both kraft HDPE–lignin blends may have additionally led to this general odor difference of the samples. Regarding specific attributes, clear correlations could be elaborated between sensory analyses and analytical characterization: the *honey-like* note corresponds with the *honey-like* smelling phenylacetic acid, 3-phenylpropanoic acid and phenylacetaldehyde, which were detected with the highest OD-factors in the pure soda lignin and its blend. A direct linkage of the *hay-like* odor with single odorous compounds is not as straightforward at first sight. However, the combination of carboxylic acids and especially of saturated and unsaturated aldehydes, together with the absence of sulfur compounds, is likely to have evoked the *hay-like* smell, which also represented the main difference between the clusters. *Hay-like* odor impressions have repeatedly been reported as off-flavors in various foodstuffs such as dried parsley, potato flakes, and peas [35,36,37]. In most of these cases, the *hay-like* smell was not caused by single odorants but rather by a complex mixture of compounds. Comparable to both soda lignin samples, diverse mono- and di-unsaturated carbonyls played an important role for the perceived *hay-like* off-odor in the food samples, which were related to the peroxidation of unsaturated fatty acids. With regard to the only difference in odor between the soda lignin and its corresponding blend, the stronger *vanilla-like* note of the pure soda lignin is related to the high OD-factor of >2187 of vanillin in this sample.

The odor impressions of the kraft HDPE–lignin blends were also comparable. Both were almost exclusively described as smelling *burnt, charcoal-like,* and *smoked ham-like/clove-like*. This correlates well with the detection of the *smoky* smelling guaiacol, which was found as the strongest perceived odorant and therefore key odor-active compound in the investigated sample material. Only in the soda HDPE–lignin blend, guaiacol was not perceived in the highest dilution, explaining the moderate ratings for the *smoked ham-like, clove-like* attribute in case of this sample. Besides guaiacol, several other *smoky* and/or *clove-like* smelling phenolic compounds were detected with high OD-factors in all blends, thus additionally contributing to the *smoked ham-like/clove-like* odor. It can be assumed that the complex mixture of these odorants also was the likely reason for the strong *burnt, charcoal-like* smell, which is in line with previous reports on *burnt* smells of wood smoke, ashtrays, diesel exhausts and cigarette smoke [38,39,40,41,42]. While diverse phenolic compounds, particularly the intense odorants guaiacol and 2,6-dimethoxyphenol [38,43,44], have previously been described as dominant contributors in smoke odor, additional phenolic trace compounds and other compound classes have also been reported as smoky constituents, namely various pyrazines, furans, and single substances such as cycloten and vanillin [39,40,41]. Many of these compounds were also detected in the investigated sample materials in this study. Interestingly, although phenolic compounds were detected with high OD-factors in the soda lignin samples, too, the *smoky* and *burnt* odor was less prominent and potentially covered by other intense odorants such as the group of aldehydes. Furthermore, in case of the kraft softwood HDPE–lignin blend, also sulfur compounds were detected with notable OD-factors, marking the most relevant difference when comparing both kraft HDPE–lignin blends. This correlated well with the sensory results, as the kraft softwood HDPE–lignin blend was the only sample that was described as having a *sulfuric* smell.

Strongly comparable to its corresponding blend, the pure kraft softwood lignin was also characterized by an intense *burnt, charcoal-like* odor impression, while a *sulfuric* note was also perceived, directly attributable to the detection of several sulfur compounds. The sole and main difference between the pure kraft softwood lignin and its corresponding blend was the significantly higher *smoked ham-like/clove-like* odor intensity in case of the pure lignin, which again correlates well with the detection of guaiacol with an extremely high OD-factor > 2187.

In contrast to the high similarity of both the soda and the kraft softwood lignins to their corresponding blends, the odor of the pure kraft pine lignin deviated considerably from its blend. Generally, the odor was described with moderate ratings for a number of attributes rather than with one or a few characteristic attributes as was the case for the other samples. Accordingly, this sample was not assignable to any of the other sample clusters. Analytical results confirmed these findings since the lowest number of odorants was detected in the kraft pine lignin, whereas only five of them reached OD-factors ≥ 243. These included guaiacol, explaining the *smoked ham-like, clove-like* odor impression, and vanillin, for the *vanilla-like* odor of the pure kraft pine lignin. Although vanillin showed notable OD-factors in all samples, it was perceived the strongest in the pure kraft pine lignin, which was likely due to the fact that it was not covered by other odorants as in the other samples. Apart from that, the alkylated 2-cyclopenten-1-ones were also detected with high OD-factors and may additionally contribute to the differentiation in the overall odor profile, despite the fact that their distinct *caramel-like* or *lovage-like* smell was not chosen as a specific odor attribute. However, such sweetish impressions might coincide with the other notes, namely *vanilla-like* or *honey-like*. Interestingly, 2,3-butanedione was not detected in the kraft pine lignin although a *butter-like* odor impression was perceived with higher ratings in comparison to the other samples.

### 4.2. Influence of Raw Materials and Processing on the Odor Profiles

#### 4.2.1. Influence of Raw Materials

Focusing on the potential origin of the detected odorants, the contribution of the virgin HDPE pellets used for the production of the blends was negligible since no odor-active compounds were perceived in the distillate of the analyzed sample aliquot. Consequently, the lignin raw material was identified as the main source of odor. In addition, the processing conditions during the production of the blends could also be confirmed as an impacting factor influencing single odorous compound classes.

**Figure 4:**
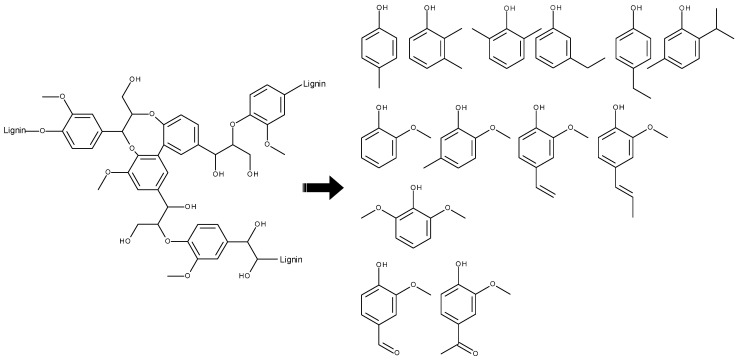
Exemplary structural motif of lignin [7,45] and aromatic degradation products which have been identified as odor-active compounds in the analyzed sample materials.

Due to the fact that lignin is a cross-linked aromatic-based heteropolymer and therefore highly heterogeneous in its naturally occurring molecular structure, numerous degradation products have been reported so far [7,45]. These include primarily aromatic compounds, especially substituted phenols and mono-/di-methoxyphenols, of which many were detected as odor-active compounds in the investigated sample material (see Figure 4). Until today, based on the dominance in gas chromatographic analyses, most often guaiacol and vanillin are held mainly responsible for odors originating from lignins, followed by structurally related compounds such as isoeugenol, syringol, vinyl guaiacol, and 4-acetyl-2-methoxyphenol (acetovanillone) [7,24,26,28,45]. This was in good accordance to the detected odorants in the present study. Additionally, several other phenolic compounds were also detected with considerable OD-factors, such as 2,6-dimethylphenol, *p*-cresol and 3-ethylphenol (no. 48, 55, 58), which are likely contributors to the off-odor perceived in the investigated lignins and corresponding HDPE blends. This is especially valid as they act as an odorant group which might lead not only to additive but even synergistic effects, as has been previously described in smell research in numerous cases [46,47].

Besides lignin-derived phenolic structures, sulfur compounds have repeatedly reported to cause odor problems, especially in the case of lignins resulting from sodium sulfide using kraft processes [24,26]. As in the case of guaiacol and vanillin, only few substances have hitherto been related to the off-odor, and only those that are detectable by conventional (headspace) GC-MS analysis, including the highly volatile methanethiol, dimethyl sulfide, and dimethyl disulfide [28]. However, utilizing the olfactometric detection, especially dimethyl tri- and tetra-sulfide with high OD-factors and many other structurally diverse sulfur containing compounds could be revealed in the present study, thus complementing our picture not only on phenolic but also on sulfur-containing potential odor contributors. As anticipated based on the sensory evaluation, the kraft softwood lignin and its corresponding blend were affected the most by the sensory impact of the sulfur compounds, thus explaining the sulfuric smell perceived by the panel. In contrast to that, the samples resulting from the sulfur-free soda process showed hardly any sulfur compounds with the sole exception of dimethyl trisulfide. Yet, this compound only gave OD-factors equal or lower than 27. Surprisingly, in the pine lignin almost no sulfur-containing odorants were detectable although resulting from a kraft process. Described as a ‘highly purified form of kraft lignin’ in the product information sheet, potential valorization of this lignin could explain the absence of sulfur-containing odor-active compounds, and therefore the exceptional sensory quality of this material.

The type of lignin isolation and depolymerization also influenced the occurrence of odorous carboxylic acids in the investigated samples. Most of these compounds were detected with higher OD-factors in the soda lignin than in both kraft lignins. It has been reported that the soda process leads to the formation of fragments with lower molecular weight when being operated under stronger oxidation conditions, among them short-chain carboxylic acids and substituted derivatives of benzoic acid [7,8]. Accordingly, free fatty acids and other volatile degradation products thereof have previously been as associated with odor problems in lignin. For example, hexanal was found in headspace analyses of lignin from wheat straw and was attributed to the autoxidation of linolenic acid [23]. This is supported by our findings; especially in the soda lignin samples, we could confirm hexanal as oxidation product, and, moreover, a multitude of other saturated and unsaturated aldehydes that had previously not been detected in other studies. These aldehydes were detected with commonly high OD-factors, whereas in the kraft lignin samples such compounds only played a minor role. The great load of the soda lignin with aldehydes implies a higher content of (un-)saturated fatty acids as precursors that presumably were entrapped in the solid structure of the raw material used for the production of this lignin. Accordingly, the base chemical composition and purity level of the lignin material is a likely quality-defining parameter with regard to smell. Such findings might call for improved purification processes in the future. Moreover, the natural source of the lignin as well as the isolation process have recently reported to not only influence the chemical composition but also the overall physicochemical properties of the lignin such as the molecular weight, the glass transition temperature, or the solubility [10]. Accordingly, processes need to be established that comply both with the techno-functional as well as sensory requirements of this valuable raw material.

#### 4.2.2. Influence of Processing

The blends of the current study contained 30% lignin. Accordingly, a direct comparison of OD-factors of odorants detected in the pure lignins and corresponding blends is limited and requires some extrapolation. In view of this, it is interesting to note that in case of carboxylic acids a substantial decrease of up to four OD-factor steps in the blends compared to the corresponding lignin raw material was observable. This indicates a degradation or loss of this odorant class during the production and/or processing of the blends. Moreover, furan-derived odorants were exclusively detected with commonly high OD-factors in the blends while being almost absent in the pure lignins (see Figure 3). Thus, a formation of such compounds during the production of the blends is likely, which is certainly associated with thermal effects since furan compounds are known to be formed by diverse thermal degradation and rearrangement processes of carbohydrates [48,49]. This especially refers to the melt temperature during the production of the blends, which was set at 195 °C in this study. The melt temperature defines the extent of the degradation of lignin, which, on the other hand, influences the formation and release of odorants. However, since both the kraft softwood lignin as well as the soda lignin were very similar in odor compared to their corresponding blend, the neo-formation of these furan compounds did obviously not affect the odor of the blends to a relevant extent. Nevertheless, it might be necessary to further control or optimize temperature effects in case of other materials and blend compositions.

Considerable differences in odor and therefore possible effects of processing were only observable in the case of the kraft pine lignin and its corresponding blend. In accordance with the specified purification, the pure kraft pine lignin showed the lowest odorant load and, correspondingly, the least intense smell, where the *burnt, charcoal-like* note was almost completely absent. In contrast to this, the *burnt* note was strongly perceivable in the kraft pine HDPE blend. The generation of a *burnt* odor during the production and processing of the blend can be attributed to the formation of additional furan-derived and sulfur compounds since this was found to be the major difference in the OD-factors of single odorant classes of the pure kraft pine lignin and its corresponding blend. Especially dimethyl trisulfide and 2-furfurylthiol, detected with OD-factors 1–3 in the pure lignin, were perceived with substantial higher OD-factors of 81–243 in the blend, indicating relevant quantitative differences. Possible reasons for this odorant generation might be non-odorous elementary sulfur or other sulfur compounds potentially remaining in the purified raw lignin and thus served as odorant precursors. Shear force- and heat-intense processing of the kraft pine HDPE–lignin blend might then release these potent odorants. Consequently, this calls for comprehensive strategies for odor control and reduction during the manufacturing of the blends.

## 5. Conclusions

The usage of the natural polymer lignin in polyolefin blends is promising in terms of reducing the need for mineral oil-based plastic materials, however, high odor load often limits further applications of such composites. Focusing on HDPE–lignin blends, objective of the present study was the detailed characterization of odorous compounds to evaluate the influence of the blend production and of different lignins used as raw material.

Sensory evaluation of three examined lignins and their corresponding HDPE blends revealed high odor intensities, which was confirmed and further elucidated by the detection of a total of 71 single odor-active compounds in the sample distillates. 90% of these odorants were identified on a molecular level and a large part thereof was reported as odorous constituents of lignin for the first time. Most of the detected odorants originated from the structurally complex lignin raw material and could be assigned to six main substance classes: carboxylic acids, aldehydes, phenols, furan compounds, alkylated 2-cyclopenten-1-ones, and sulfur compounds.

The natural source of the lignin as well as the isolation process showed strong influences on the composition and content of these odorous substances in the investigated materials. The kraft softwood lignin as well as both kraft HDPE–lignin blends were primarily characterized by *smoky* and *burnt* odors evoked by diverse phenolic and sulfur compounds, resulting from the sulfurous kraft process. Due to high purification, the kraft pine lignin, however, showed a comparably lower odor load with a less pronounced character of the odor profile. In contrast, the odor profiles of the soda lignin and its corresponding blend were described as *hay-* and *honey-like*, being a result of a complex mixture of odorants, and were thus clearly distinguishable from the samples resulting from kraft processes. As in case of the kraft lignin samples, phenolic compounds accounted for the main number of odorous substances, however, carboxylic acids and a number of saturated and unsaturated aldehydes were additionally detected in the soda lignin samples. The latter stem from degradation of fatty acids and were presumably entrapped as impurities in the original soda lignin structure.

Besides, the blend production and processing partially influenced the odor as neo-formation of odorants occurred mainly in the case of furan derivatives. However, additional sulfur compounds were detected in the kraft pine HDPE–lignin blend which were absent in the pure lignin, being further potential contributors to the *burnt* odor impression. From this study we conclude that the selection of the lignin raw material and optimized purification protocols as well as odor reduction strategies during the compounding process itself are of prime importance in the development of odor-optimized HDPE–lignin blends.

## Figures and Tables

**Table 1:** Details and characteristics of raw materials used for the production of the HDPE–lignin blends.

Sample Name	Supplier	Commercial Name	Characteristics According to the Supplier’s Specification
virgin HDPE	Sabic	HDPE M80064	injection molding grade; narrow molecular weight distribution; MFR = 8.0 g/10 min
compatibilizer	DuPont	Fusabond E-MB 100D	maleic anhydride grafted HDPE; MFR = 2.0 g/10 min
soda lignin	PLT Innovations	Protobind 1000	sulfur free (>90%) soda lignin from agricultural fibrous feedstocks (wheat straw/Sarkanda grass)
kraft pine lignin	Ingevity	Indulin AT	kraft softwood lignin resulting from pinewood only; highly purified; free of all hemicellulose materials
kraft softwood lignin	UPM Biochemicals	BioPiva 100	kraft softwood lignin resulting from different softwoods, sulfur content < 3%

**Table 2:** Odorants identified in distillates of the pure lignins and their corresponding HDPE blends, along with the OD-factors determined via OEDA and identification criteria.

No. ^a^	Odorant	Odor Quality ^b^	RI ^c^ on		OD ^d^						
			DB-FFAP	DB-5	Soda Lignin		Kraft Pine Lignin		Kraft Softwood Lignin		Identification Criteria ^e^
					HDPE-Lignin Blend	Pure Lignin	HDPE-Lignin Blend	Pure Lignin	HDPE-Lignin Blend	Pure Lignin	
1	2,3-butanedione	butter-like	984	601	27	1	1	<1	1	<1	RI, O, MS, RC
2	2,3-pentanedione	butter-like	1056	698	1	<1	<1	<1	<1	<1	RI, O, RC
3	dimethyl disulfide	cabbage-like	1077	708	<1	<1	<1	<1	1	9	RI, O, MS, RC
4	hexanal	grassy	1080	801	1	27	<1	<1	<1	<1	RI, O, MS, RC
5	unknown	sulfurous	1113	n.d.	1	<1	1	<1	1	1	-
6	4-methoxy-2-methyl-2-butanethiol	blackcurrant-like	1206	925	<1	<1	<1	<1	9	3	RI, O, RC
7	unknown	garlic-like, sulfurous	1239	n.d.	<1	<1	<1	1	<1	3	-
8	(*Z*)-4-heptenal	fishy, fatty	1231	896	9	27	<1	<1	<1	<1	RI, O, MS, RC
9	bis(methylthio)methane	sulfurous, garlic-like	1271	898	<1	<1	<1	<1	9	9	RI, O, MS, RC
10	octanal	citrus-like, soapy	1281	1002	1	3	<1	<1	<1	<1	RI, O, MS, RC
11	1-octen-3-one	mushroom-like	1292	979	3	9	<1	<1	<1	<1	RI, O, RC
12	1-methoxy-3-methyl-3-pentanethiol	blackcurrant-like	1324	1036	<1	<1	1	<1	9	<1	RI, O
13	2-acetyl-1-pyrroline	popcorn-like, roasty	1327	928	1	3	1	1	<1	<1	RI, O, RC
14	dimethyl trisulfide	garlic-like, cabbage-like	1365	970	27	9	243	3	81	81	RI, O, MS, RC
15	4-mercapto-4-methyl-2-pentanone	blackcurrant-like, sulfurous	1374	943	<1	<1	<1	<1	<1	9	RI, O, RC
16	nonanal	citrus-like, soapy	1383	1103	<1	1	<1	<1	<1	<1	RI, O, MS, RC
17	1,1-bis(ethylthio)ethane	sulfurous	1387	1082	<1	<1	<1	<1	<1	27	RI, O
18	(*E*)-2-octenal	fatty	1418	1057	1	27	<1	<1	<1	<1	RI, O, MS, RC
19	2-furfurylthiol (2-furanmethanethiol)	roasted coffee bean-like	1428	914	81	3	81	1	243	27	RI, O, MS, RC
20	acetic acid	vinegar-like	1445	619	3	27	1	27	<1	3	RI, O, MS, RC
21	2-ethyl-3,5-dimethylpyrazine	earthy	1449	1095	1	9	3	<1	<1	<1	RI, O, MS, RC
22	(*E,E*)-2,4-heptadienal	fatty, cucumber-like	1483	1016	<1	1	<1	<1	<1	<1	RI, O, MS, RC
23	(*Z*)-2-nonenal	green, fatty	1493	1145	3	27	3	3	9	1	RI, O, MS, RC
24	(*E*)-2-nonenal	fatty, cardboard-like	1523	1160	81	729	9	3	81	1	RI, O, MS, RC
25	3,5-dimethyl-2-vinylpyrazine	earthy	1548	1109	3	<1	1	<1	<1	<1	RI, O, RC
26	(*E,Z*)-2,6-nonadienal	cucumber-like	1573	1159	27	243	9	9	3	<1	RI, O, MS, RC
27	unknown	blackcurrant-like	1600	n.d.	1	<1	3	<1	9	9	-
28	2-acetylpyrazine	popcorn-like, roasty	1610	1023	1	1	3	<1	3	1	RI, O, RC
29	butanoic acid	cheesy, sweaty	1618	808	1	27	1	9	<1	3	RI, O, MS, RC
30	4-mercapto-2-butanol	leek-like, burnt, onion-like	1623	919	<1	<1	3	<1	9	1	RI, O, RC
31	phenylacetaldehyde	honey-like, flowery	1638	1050	9	3	<1	<1	1	1	RI, O, MS, RC
32	2-/3-methylbutanoic acid	cheesy, sweaty	1650	870	1	81	<1	3	<1	9	RI, O, MS, RC
33	2-methyl-3-(methylthio)furan	meat-like, cabbage-like	1656	943	3	3	3	<1	9	1	RI, O
34	2-methyl-3-(methyldithio)furan	broth-like, meat-like	1667	1178	27	<1	81	<1	81	1	RI, O
35	unknown	coriander-like, geranium-like	1689	1290	27	81	<1	<1	<1	<1	-
36	(*E,E*)-2,4-nonadienal	fatty, nutty	1692	1212	9	81	1	<1	3	1	RI, O, MS, RC
37	pentanoic acid	cheesy, fruity	1725	894	<1	9	<1	1	<1	1	RI, O, MS, RC
38	2-acetyl-2-thiazoline	popcorn-like, roasty	1735	1108	<1	<1	<1	<1	<1	3	RI, O, RC
39	dimethyl tetrasulfide	sulfurous, cabbage-like	1738	1223	1	<1	27	<1	27	<1	RI, O, MS
40	(*E*)-2-undecenal	coriander-like	1744	1365	27	27	9	3	9	3	RI, O, MS, RC
41	3-methylpentanoic acid	cheesy, fruity	1777	950	<1	1	<1	1	<1	<1	RI, O, RC
42	3-ethyl-2-hydroxy-2-cyclopenten-1-one	caramel-like	1792	1053	27	2187	9	243	27	2187	RI, O, MS, RC
43	(*E,E*)-2,4-decadienal	fatty	1801	1317	3	1	3	<1	9	<1	RI, O, MS, RC
44	*(E*)-*β*-damascenone	grape juice-like	1809	1387	3	27	<1	<1	<1	<1	RI, O, MS, RC
45	cycloten (2-hydroxy-3-methyl-2-cyclopenten-1-one)	lovage-like	1827	1029	81	243	27	243	27	729	RI, O, MS, RC
46	unknown	caramel-like	1850	1142	81	729	27	243	27	729	-
47	2-methoxyphenol (guaiacol)	smoky, smoked ham-like	1862	1087	243	2187	2187	2187	2187	>2187	RI, O, MS, RC
48	2,6-dimethylphenol	rubber-like	1904	1106	<1	3	9	3	9	27	RI, O, MS, RC
49	2-phenylethanol	rosy, flowery	1908	1115	3	27	<1	<1	1	81	RI, O, MS, RC
50	2-methoxy-5-methylphenol	smoky, clove-like	1935	1191	1	1	<1	<1	1	3	RI, O, MS, RC
51	5-methylhexanoic acid	cheesy, sweaty	1957	1020	<1	<1	<1	<1	<1	9	RI, O, RC
52	unknown	broth-like, meat-like	1963	n.d.	81	<1	729	<1	729	<1	-
53	4-hydroxy-2,5-dimethyl-3(2H)-furanone (furaneol)	caramel-like	2022	1076	243	3	27	<1	81	1	RI, O, MS, RC
54	*γ*-nonalactone	coconut-like	2026	1360	<1	27	1	81	<1	81	RI, O, MS, RC
55	4-methylphenol (*p*-cresol)	horse stable-like, fecal	2078	1068	3	27	3	3	3	9	RI, O, MS, RC
56	2,3-dimethylphenol	phenolic, leather-like	2141	1200	<1	1	<1	<1	1	3	RI, O, MS, RC
57	4-ethylphenol	fecal, phenolic	2169	1171	<1	<1	<1	<1	<1	3	RI, O, MS, RC
58	3-ethylphenol	leather-like, phenolic	2172	1179	27	729	3	9	3	27	RI, O, MS, RC
59	2-isopropyl-5-methylphenol (thymol)	thyme-like	2178	1297	<1	1	<1	<1	3	9	RI, O, MS, RC
60	2-methoxy-4-vinylphenol	clove-like, smoked ham-like	2195	1326	2187	243	729	1	81	3	RI, O, MS, RC
61	wine lacton	coconut-like, dill-like	2213	1458	<1	<1	3	3	9	27	RI, O, RC
62	2,6-dimethoxyphenol	smoked ham-like, smoky	2260	1363	243	2187	27	81	3	9	RI, O, MS, RC
63	cinnamyl alcohol	flowery	2287	1313	<1	<1	<1	1	<1	27	RI, O, MS, RC
64	isoeugenol	clove-like, smoky	2311	1453	9	<1	27	1	9	<1	RI, O, MS, RC
65	*γ*-dodecalactone	peach-like	2374	1679	27	81	1	3	1	1	RI, O, MS, RC
66	unknown	smoky, clove-like	2452	n.d. ^f^	9	<1	9	3	9	<1	-
67	3-methylindole (skatole)	fecal, mothball-like	2496	1391	9	9	9	<1	<1	<1	RI, O, MS, RC
68	phenylacetic acid	honey-like, beeswax-like	2541	1254	27	243	<1	3	<1	27	RI, O, MS, RC
69	vanillin	vanilla-like	2563	1400	2187	>2187	243	729	243	243	RI, O, MS, RC
70	3-phenylpropanoic acid	honey-like, flowery	2626	1339	1	9	<1	1	<1	1	RI, O, MS, RC
71	4-acetyl-2-methoxyphenol	vanilla-like	2640	1486	27	729	9	3	1	27	RI, O, MS, RC

^a^ Odorants consecutively numbered according to their elution on capillary column DB-FFAP. ^b^ Odor quality perceived at the odor detection port. ^c^ Retention indices (RI) on capillary columns DB-FFAP and DB-5 according to Van den Dool and Kratz [33]. ^d^ Odor dilution factor (OD) on capillary column DB-FFAP according to Grosch [31]. ^e^ Identification of odorants based on retention index (RI), odor quality (O), and mass spectrum (MS) and comparison of respective data with a reference compound (RC) if applicable. ^f^ n.d.—not detected.

## Data Availability

The data presented in this study are available on request from the corresponding author.

## Appendix A

### Chemicals and Reference Substances

The solvents pentane and dichloromethane (Th. Geyer GmbH und Co. KG, Renningen, Germany) were freshly distilled prior to use to obtain the highest purity. Liquid nitrogen was obtained from Linde Gas (Pullach im Isartal, Germany). A homologues series of linear alkanes (C_6_-C_26_) in pentane enabled the determination of retention indices in the course of gas chromatographic analyses (Fluka, Steinheim, Germany and Sigma-Aldrich, Steinheim, Germany).

Reference compounds used for the unequivocal identification of odorants are listed in the following. Suppliers and trivial names (if existing) are given in brackets.

Reference compounds obtained from Sigma-Aldrich (Steinheim, Germany): acetic acid ≥ 99%, 4-acetyl-2-methoxyphenol ≥ 98%, 2-acetylpyrazine ≥ 99%, (*E*)-*β*-damascenone ≥ 99%, (*E,E*)-2,4-decadienal ≥ 85%, 2,6-dimethoxyphenol ≥ 99%, 2,3-dimethylphenol ≥ 99%, dimethyl trisulfide ≥ 98%, 2-ethyl-3,5-dimethylpyrazine ≥ 95%, 2-furfurylthiol ≥ 98%, (*E,E*)-2,4-heptadienal ≥ 88%, (*Z*)-4-heptenal ≥ 98%, hexanal ≥ 98%, 2-hydroxy-2,5-dimethyl-3(2H)-furanone (furaneol) ≥ 99%, 2-hydroxy-3-methyl-2-cyclopenten-1-one (cycloten) ≥ 98%, 2-methoxyphenol (guaiacol) ≥ 99%, 2-methoxy-4-vinylphenol ≥ 98%, 2-methylbutanoic acid ≥ 98%, 3-methylbutanoic acid ≥ 99%, 3-methylindole (skatole) ≥ 98%, 4-methylphenol (p-cresol) ≥ 99%, (*E,E*)-2,4-nonadienal ≥ 85%, (*E,Z*)-2,6-nonadienal ≥ 95%, *γ*-nonalactone ≥ 98%, (*E*)-2-nonenal ≥ 97%, (*Z*)-2-nonenal ≥ 90%, octanal ≥ 99%, (*E*)-2-octenal ≥ 94%, 1-octen-3-one ≥ 50%, 2,3-pentanedione ≥ 97%, phenylacetaldehyde ≥ 90%, phenylacetic acid ≥ 99%, 2-phenylethanol ≥ 99%, 3-phenylpropanoic acid ≥ 99%, 3-phenyl-2-propen-1-ol (cinnamyl alcohol) ≥ 98%, and (*E*)-2-undecenal ≥ 90%.

2,3-butanedione ≥ 99%, butanoic acid ≥ 99.5%, dimethyl disulfide ≥ 98%, 2,6-dimethylphenol ≥ 99%, 2-isopropyl-5-methylphenol (thymol) ≥ 99%, 3-methylpentanoic acid ≥ 97%, 2-methoxy-4-propenylphenol (isoeugenol) ≥ 98%, nonanal ≥ 95% and pentanoic acid ≥ 99% were obtained from Fluka (Steinheim, Germany). Other reference compounds were: 2-acetyl-1-pyrroline (1-(3,4-dihydro-2H-pyrrol-5-yl)ethanone, ≥ 95%, 3,5-dimethyl-2-vinylpyrazine ≥ 95% and (3S,3aS,7aR)/(3R,3aR,7aS)-3a,4,5-7a-tetrahydro-3,6-dimethylbenzofuran-2(3H)-one (wine lactone) ≥ 98% from aromaLAB AG, Freising, Germany), *γ*-dodecalactone ≥ 97% from SAFC (Steinheim, Germany), 4-methoxy-2-methyl-2-butanethiol of unknown purity from Takasago (Zülpich, Germany), bis(methylthio)methane ≥ 99% from Alfa Aesar (Kandel, Germany), 4-hydroxy-3-methoxybenzaldehyde (vanillin) ≥ 99%, 4-mercapto-4-methyl-2-pentanone ≥ 98% and 2-methoxy-5-methylphenol of unknown purity from ABCR (Karlsruhe, Germany), 2-acetyl-2-thiazoline ≥ 98% from Chemos (Altdorf, Germany), 3-ethyl-2-hydroxy-2-cyclopenten-1-one ≥ 97% from TCI (Eschborn, Germany), 5-methylhexanoic acid ≥ 97% from Matric Scientific (W. Columbia, South Carolina), 3-ethylphenol ≥ 98% and 4-ethylphenol ≥ 99% from Riedel-de-Haen (Seelze, Germany).
